# Montelukast Influence on Lung in Experimental Diabetes

**DOI:** 10.3390/medicina60050749

**Published:** 2024-04-30

**Authors:** Cristina Gales, Bogdan Stoica, Gabriela Rusu-Zota, Mihai Nechifor

**Affiliations:** 1 Department of Histology, “Gr T Popa” University of Medicine and Pharmacy, Universitatii 16, 700115 Iasi, Romania; cgales81@yahoo.com; 2 Department of Biochemistry, “Gr T Popa” University of Medicine and Pharmacy, Universitatii 16, 700115 Iasi, Romania; 3 Department of Pharmacology, “Gr T Popa” University of Medicine and Pharmacy, Universitatii 16, 700115 Iasi, Romania; gabrielarusu1105@gmail.com

**Keywords:** diabetes, lung fibrosis, leukotrienes, montelukast

## Abstract

*Background and Objectives*: The influence of montelukast (MK), an antagonist of cysLT1 leukotriene receptors, on lung lesions caused by experimental diabetes was studied. *Materials and Methods*: The study was conducted on four groups of six adult male Wistar rats. Diabetes was produced by administration of streptozotocin 65 mg/kg ip. in a single dose. Before the administration of streptozotocin, after 72 h, and after 8 weeks, the serum values of glucose, SOD, MDA, and total antioxidant capacity (TAS) were determined. After 8 weeks, the animals were anesthetized and sacrificed, and the lungs were harvested and examined by optical microscopy. Pulmonary fibrosis, the extent of lung lesions, and the lung wet-weight/dry-weight ratio were evaluated. *Results*: The obtained results showed that MK significantly reduced pulmonary fibrosis (3.34 ± 0.41 in the STZ group vs. 1.73 ± 0.24 in the STZ+MK group *p* < 0.01) and lung lesion scores and also decreased the lung wet-weight/dry-weight (W/D) ratio. SOD and TAS values increased significantly when MK was administered to animals with diabetes (77.2 ± 11 U/mL in the STZ group vs. 95.7 ± 13.3 U/mL in the STZ+MK group, *p* < 0.05, and 25.52 ± 2.09 Trolox units in the STZ group vs. 33.29 ± 1.64 Trolox units in the STZ+MK group, respectively, *p* < 0.01), and MDA values decreased. MK administered alone did not significantly alter any of these parameters in normal animals. *Conclusions*: The obtained data showed that by blocking the action of peptide leukotrienes on cysLT1 receptors, montelukast significantly reduced the lung lesions caused by diabetes. The involvement of these leukotrienes in the pathogenesis of fibrosis and other lung diabetic lesions was also demonstrated.

## 1. Introduction

Diabetes mellitus (DM) is an important public health problem everywhere in the world and one of the most serious diseases in human pathology [1].

This disease affects all organs in the human body and causes severe complications.

Pulmonary function is impaired in DM. Different studies have shown that a series of pulmonary functional parameters such as forced expiratory volume in 1 s (FEV1), total lung capacity, forced vital capacity (FVC), and other functional parameters are reduced in patients with DM compared to normal subjects of the same age [2,3,4,5]. DM impairs gas exchange and affects pulmonary microcirculation by increasing the thickness of the walls of the pulmonary capillary vessels [6].

Peptide leukotrienes (LTC4, LTD4, and LTE4) are a group of eicosanoids derived from arachidonic acid. This polyunsaturated acid is released from the phospholipids of cell membranes under the action of phospholipase A2. 

Under the action of 5-lipoxygenase (5-LO), arachidonic acid is transformed into LTA4, which is transformed into LTB4 and separated into LTC4 [7,8]. LTC4 is synthesized by the conjugation of LA4 with reduced glutathione under the action of the LTC4 synthase. From LTC4, LTD4 and LTE4 are derived. The three previously mentioned leukotrienes are called peptide leukotrienes.

Peptide leukotrienes are synthesized not only at the level of lung tissues but also at the level of macrophages, eosinophils, and basophils in the lungs [9].

Leukotrienes have two types of receptors: receptors for LTB4 and cys LT1 and cys LT2 receptors for peptide leukotrienes (LTC4, LTD4, and LTE4). All leukotriene receptors are membrane receptors coupled to G proteins. Both peptide leukotrienes and their receptors are found at the level of all tissues in the structure of the lungs and airways [10].

Peptide leukotrienes have numerous actions at the bronchopulmonary level. They are powerful proinflammatory factors and they increase the synthesis of proinflammatory cytokines, causing a strong bronchoconstriction and vasoconstriction of the pulmonary vessels.

All leukotrienes are involved in the pathogenesis of various diseases in humans (asthma, asthmatic bronchitis, atopic dermatitis, allergic conjunctivitis, and others) [11,12].

Montelukast (MK) is a selective competitive antagonist of cysLT1 receptors. MK and other cys LT1 antagonists are used in the treatment of bronchial asthma, asthmatic bronchitis, allergic conjunctivitis, and other diseases [13]. The purpose of this study was to show the action of montelukast on the lungs in experimental diabetes in rats.

## 2. Material and Methods

The study was carried out on four groups of 6 adult male Wistar rats weighing between 230–260 g kept in normal laboratory conditions (room temperature: 21–23 °C; relative humidity: 40–60%; 12-h light–dark cycle), having free access to standard rodent food and water. Animals were obtained from the Laboratory Animal Center of Cantacuzino Institute of Research, Bucharest, Romania were housed in groups of five in Plexiglas cages (65 × 40 × 30 cm) with the floor covered with sawdust.

The first group received only a 2 mL/kg/day saline solution daily for 8 weeks.

The second group received 15 mg/kg/day daily (MK) (Actavis Malta) by endogastric probe for 8 weeks.

The third group received on the first day 65 mg/kg streptozotocin (STZ) (Sigma Chemical, St. Louis, MO, USA) dissolved to 0.1 M, pH 4.5, ip. in a single dose [14].

The fourth group received on the first day 60 mg/kg STZ dissolved to 0.1 M, pH 4.5 ip. in a single dose and 15 mg/kg/day daily MK by endogastric probe for 8 weeks.

Rats were classified as diabetic if their fasting blood glucose levels exceeded 250 mg/dl. On the first day of the experiment, before the administration of any substance, all animals were weighted using an electronic scale. The weight of the rats was determined every other day, and the dose of MK was correlated with the weight.

### 2.1. Histopathological Analysis

After eight weeks all animals were anesthetized with thiopental 40 mg/kg ip. and were sacrificed by carotid sectioning. The lungs were removed from all animals. 

Both lungs from each animal were fixed in 10% neutral buffered formalin at 4 °C. After 24 h, all tissues were embedded in paraffin. A fifth section from both organs from each animal was performed with a Microtome SLEE CUT 6062 (SLEE Medical GmbH, Nieder-Olm, Germany). All sections were stained with hematoxylin and eosin for 30 min at room temperature. Masson’s trichrome staining was used to evaluate fibrosis. Both lungs of all animals were weighed after slaughter, and after that, both lungs of each animal were examined with optical microscopy using an Optika microscope (Optika, Ponteranica, Italy 2004). Six images per section were examined. 

For each lung, 5 sections were examined and for each section, 6 images were examined by two independent observers.

A semi-quantitative method was used to evaluate pulmonary fibrosis [15,16]. For each section, 6 images were examined.

The following criteria were used to grade pulmonary fibrosis:0—normal lung; 1—minimal fibrosis thickening of alveolar or bronchiolar walls; 2—moderate thickening of walls without obvious damage to lung architecture; 3—increased fibrosis with definite damage of lung structure and formation of fibrosis bands or small fibrosis masses; 4—severe distortion of structure and large fibrous areas; 5—total fibrosis obliteration of the field.

The extent of lung lesions (fibrosis and neutrophil infiltration) was evaluated with the following score [17]: 0 = none; 1 = lesions involving <0–25% of the lung, (mild severity); 2 = lesions involving <25–50% of the lung (moderate severity); 3 = lesions involving >50% of the lung (severe).

Pulmonary tissue edema was evaluated by the determination of the lung wet-weight/dry-weight ratio (W/D) [18]. For the determination of the lung W/D, after the rat sacrifice, the middle lobes from both lungs were removed. Only the middle pulmonary lobes were weighed with a balance after being placed on filter paper, and the weight was recorded for each lobe. Afterward, the lobes were dried by exposure to 80 C heat for 72 h. After this, the middle lobes were weighed again, and the W/D ratio was determined [19].

### 2.2. Biochemical Analysis

On the first day of the experiment (before the administration of STZ), blood was collected by venipuncture (from the tail), and the levels of plasma glucose, malondialdehyde (MDA), superoxide dismutase (SOD), and total antioxidant status (TAS) were determined.

At 72 h and 8 weeks after the start of the experiment, the blood collection was repeated, and the same biochemical analyses were repeated in all animals.

Blood samples from the tail were taken from each animal and were centrifuged at 6500 for 10 min, and the plasma was stored at −20 °C until analysis. Serum glucose levels were measured by a spectrophotometric method using a Randox Daytona UK analyzer. 

Superoxide dismutase SOD (natural antioxidant enzyme) is an important antioxidant marker with protective cellular action against oxidative stress. This enzyme converts the superoxide anion to H_2_O_2_. A colorimetric method was used to determine SOD activity and the colorimetric monitoring of superoxide anion formation using a kit for SOD [20]. Kits and reagents used were produced by Randox Laboratories Ltd., Crumlin, UK. 

Increased lipid peroxidation is one of the most important pathogenic mechanisms of damage production in diabetes mellitus. Malondialdehyde (MDA) is an important secondary product resulting from lipid peroxidation. Determining the concentration of this substance is considered important as a biomarker of oxidative stress. The level of the lipid peroxidation product MDA was determined spectrophotometrically. MDA was tested using a thiobarbituric acid (TBA) assay. Through the reaction between MDA and TBA, a conjugate is formed that absorbs light in the visible spectrum at 535 nm and produces a red-pink color [21].

Total antioxidant status was measured in serum as a Trolox Equivalent Antioxidant Capacity (TEAC), [22]. The total antioxidant status (TAS) was assayed with a chemiluminometric method using the luminol–horseradish peroxidase system (a Berthold Lumat 9507 chemiluminometer, Berthold, Bad Wildbad, Germany was used). In this method, constant light emission results from luminol degradation in the presence of a catalyst (horseradish peroxidase) with an enhancer (p-iodo-phenol) and is recorded kinetically. When a biological fluid is introduced into this system, the level of light emission decreases for a period; this is proportional to the total antioxidant capacity. The principle of the antioxidant assay (TAS determination) is the formation of a ferryl myoglobin radical from metmyoglobin and hydrogen peroxide, which oxidizes the ABTS (2,2¢-azino-bis(3-ethylbenzthiazoline-6-sulfonic acid) to produce a radical cation, ABTS·+, a soluble chromogen that is green in color and can be determined spectrophotometrically. After adding 150 µL of ABTS substrate working solution to each well, the incubation time was 5 min at room temperature.

Trolox (6-hydroxy-2,5,7,8-tetramethylchroman-2-carboxylic acid), a water-soluble, alpha-tocopherol analog, was used as the standard. Calibration was performed with Trolox (hydro-soluble vitamin E) (Sigma Aldrich, St. Louis, MO, USA), and the final results relate to Trolox equivalents. The pro-oxidant system, which generates light, was brought to five million relative units of light (RLU), and serum samples were used at a dilution of 1/10 [23]. This method determines the antioxidative effect and results are expressed in Trolox units. The Antioxidant Assay Kit-CS0790 from Sigma-Aldrich, St. Louis, MO, USA was used to quantify TAS.

### 2.3. Statistical Interpretation of the Data

The obtained data were statistically interpreted by a one-way ANOVA test implemented in the SPSS program for Windows 10 Analytics version 17.0 software. Data are given as mean ± SD. A value of *p* < 0.05 was considered significant. To assess normality, we used the Shapiro–Wilk test from the SPSS program (with a *p* value greater than 0.05). To assess homoscedasticity, we used the linear regression statistics from the SPSS program (with the scatterplot of the residuals equally distributed).

The research was conducted after obtaining the agreement of the Ethics Committee of Research of “Gr. T Popa” University of Medicine and Pharmacy and agreed with the EU directive 2010/63/EU regarding the handling of laboratory animals. 

## 3. Results 

The obtained results showed that MK had a partially protective action against lung lesions induced by experimental diabetes.

Diabetes significantly increased blood glucose levels in all diabetic animals included in the study. In all animals from groups three and four, the plasma glucose level was higher than 250 mg/dL. MK administered alone did not change blood glucose values. The administration of MK to the animals that received STZ did not significantly change the plasma glucose level at 72 h (346.5 ± 11.4 mg/dL in the STZ group vs. 337.7 ± 10.2 mg/dL in the STZ+MK group) and slightly decreased it at 8 weeks (359.1 ± 6.7 mg/dL in the STZ group vs. 322.8 ± 7.2 mg/dL in the STZ+MK group, *p* < 0.05). 

STZ diabetes significantly decreased TAS values. MK administered alone did not change TAS, and the administration of MK to diabetic animals significantly increased TAS compared to animals that received only STZ (Table 1).

The increased values of MDA after STZ administration were significantly reduced by MK (Table 2).

SOD was low in animals that received only STZ. MK administered to animals that received STZ significantly increased SOD (Table 3).

The weights of the rats in groups I and II did not significantly change during the 8 weeks of the experiment (249.3 ± 7.3 g before the treatment and 256.1 ± 8.5 g after 8 weeks in the first group and 251.2 ± 9.4 g after 8 weeks). The group that received only STZ had a significantly lower weight compared to the initial weight (253 ± 5.7 g before the experiment and 181.7 g ± 8.9 g after 8 weeks, *p* < 0.01). MK significantly reduced the weight loss in diabetic rats (253.9 ± 6.8 g before the experiment vs. 224.1 ± 7.5 g after 8 weeks in the four groups). The difference between the weight losses of groups three and four was statistically significant (*p* < 0.05).

**Figure 1:**
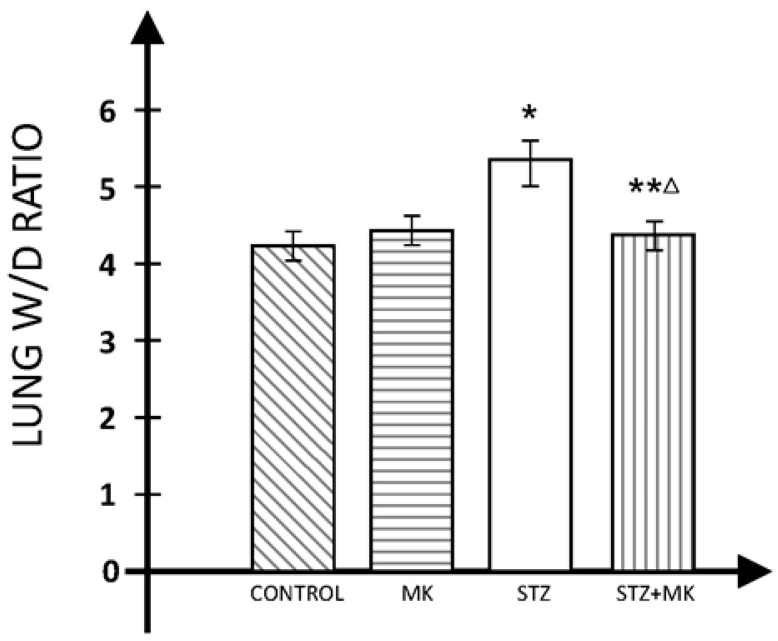
Lung W/D ratio. * *p* < 0.01 versus control group, ** *p* < 0.05 versus control group, Δ *p* < 0.05 versus STZ group.

The W/D ratio was significantly higher in diabetic animals than in the control group. MK reduced that increase in the W/D ratio in diabetic animals. There was no difference between W/D in the lungs of animals that received only MK compared to the control group (Figure 1).

**Figure 2:**
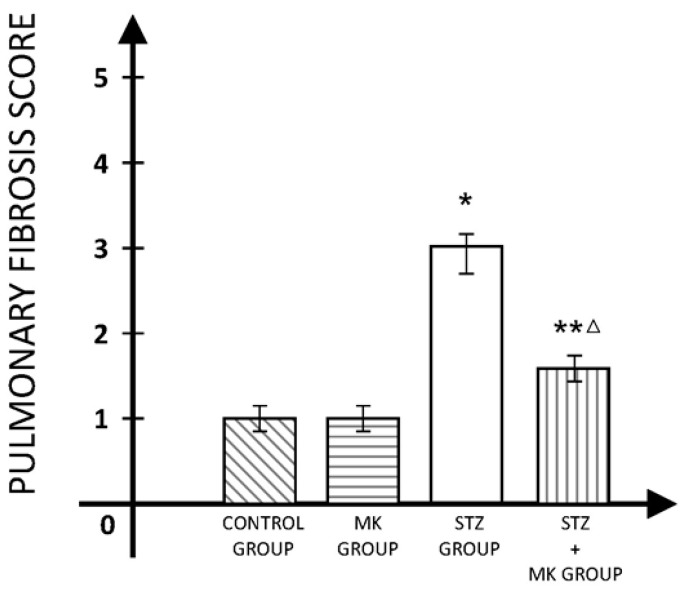
Pulmonary fibrosis score. * *p* < 0.01 versus control group, ** *p* < 0.05 versus control group, Δ *p* < 0.01 versus STZ group.

Pulmonary fibrosis was significantly more developed in diabetic animals compared to those in the control group. MK statistically significantly reduced the fibrosis score (3.34 ± 0.41 in the STZ group vs.1.73 ± 0.24 in the STZ+MK group, *p* < 0.01) (Figure 2).

**Figure 3:**
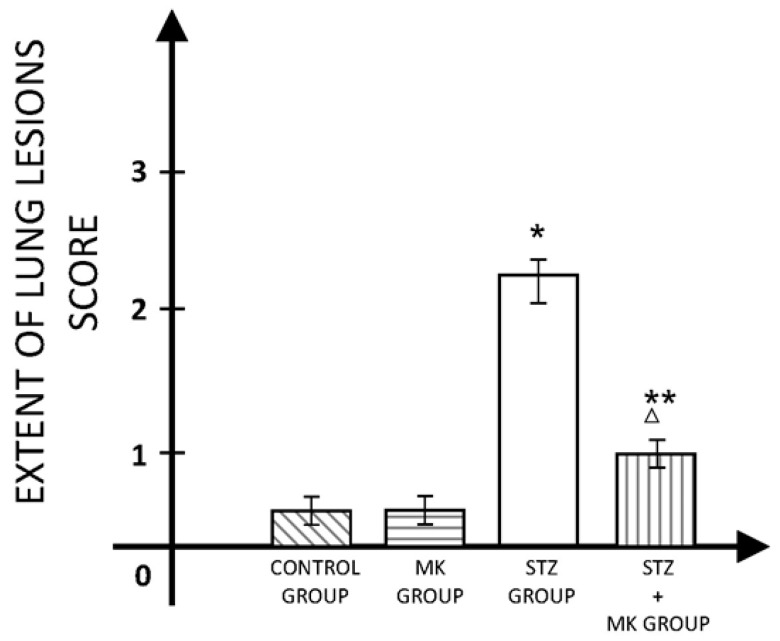
The extent of lung lesions’ score. * *p* < 0.01 versus control group, ** *p* < 0.05 versus control group, Δ *p* < 0.01 versus STZ group.

The extent of lung lesions was significantly higher in animals with STZ diabetes compared to the control group but also compared to the group that received MK after STZ administration (Figure 3).

Montelukast influence on lung tissue in rats with diabetes is shown in the following figures (Figures S1–S6). Figure S1. Control group, normal lung tissue (×100). Figure S2. Control group, with thin alveolar walls, uniform in size and shape (×400). Figure S3. STZ group (after 8 weeks), perivascular inflammatory infiltration (×100). Figure S4. STZ group (after 8weeks), alveolar septum thickening, septal inflammatory cell infiltration (×400). Figure S5. MK+STZ group (after 8 weeks), the alveolar septum thickening was significantly reduced (×100). Figure S6. MK+STZ group (after 8 weeks), reduced scattered interstitial haemorrhage, reduced thickening of the alveolar septum. 

## 4. Discussions

DM produces numerous morphological and functional changes at the pulmonary level. The amount of collagen increases, the extracellular matrix also increases, and the alveolar space decreases. An increase in the thickness of the alveoli-capillary membrane occurs [24].

In human diabetes but also STZ diabetes, the level of proinflammatory cytokines is increased.

The implications of peptide leukotrienes in pulmonary pathology are multiple and are not limited to their role in bronchial asthma bronchoconstriction and asthmatic bronchitis. Pulmonary inflammation caused by respiratory syncytial virus infection was reduced by inhibition of leukotriene synthesis with zileuton [25]. The cysteinyl (cys) leukotrienes are involved in allergen-induced airway eosinophilia and the administration of MK reduces this process [26]. In an experimental study carried out on adult rabbits exposed to an explosion of open air, a significant increase in the synthesis of peptide leukotrienes occurred in the lung. The inhibition of the pulmonary synthesis of leukotrienes determined in that case the reduction in pulmonary edema [27]. One of the complications that occur after lung transplantation or hematopoietic stem cell transplantation is bronchiolitis obliterans syndrome. MK reduces the severity of this syndrome [28]. A deterioration of pulmonary function is sometimes encountered after cardiopulmonary bypass. The factors involved in the pathogenesis of this complication are multiple and still insufficiently known. One of these factors is the significant increase in the synthesis of peptide leukotrienes [29].

Peptide leukotrienes and LTB4 are important proinflammatory agents [30]. The proinflammatory action of LTC4, LTD4, and LTE4 is exerted directly (increasing vascular permeability, increasing free radical starvation) but indirectly by stimulating the secretion of proinflammatory cytokines (IL-6, TNF alpha) [31,32]. These eicosanoids are involved in the production of various types of pulmonary fibrosis.

Peptide leukotrienes are involved in the development of experimental pulmonary fibrosis. LTC4 binds to the cys LT1 receptors on lung fibroblasts and stimulates collagen production.

LTD4 amplifies the effect of fibronectin in stimulating the migration of lung fibroblasts. This effect is mediated by the stimulation of cysLT1 receptors and is blocked by pranlukast, which, like MK, is a selective competitive antagonist of cysLT1 receptors [33].

These lipids increase the synthesis and deposition of collagen and therefore determine the development of pulmonary fibrosis. This fact is associated with an increased level of IL-4/-13 and TGF-β in the lungs [34]. The fibroblasts’ secretion of leukotrienes is increased in the lungs of elderly people, and this is one of the factors that determine the development of pulmonary fibrosis [35,36]. In different forms of pulmonary fibrosis, such as post-irradiation fibrosis, the concentration of LTC4, LTD4, and LTE4 is increased. The reduction in the level of peptide leukotrienes is associated with a reduction in experimental pulmonary fibrosis post-irradiation [37]. 

Some authors have shown that MK has the potential to be used to improve the condition of patients with cystic fibrosis and other lung diseases due to its action of reducing the accumulation of collagen in the lung.

Along with other factors, peptide leukotrienes are also involved in silica-induced pulmonary fibrosis in mice [38]. Our data showed that MK significantly reduced the development of diabetic pulmonary fibrosis and decreased the infiltration of diabetic lungs with neutrophils. MK administered alone in normal rats did not change pulmonary fibrosis or lung lesions’ score. Other studies have shown that in STZ diabetes, there is an increase in pulmonary infiltration with neutrophils and macrophages and an increase in the secretion of proinflammatory cytokines (IL-1β, IL-6) [39,40]. TGF-1beta is also important in diabetes-induced fibrosis [41]. LTD4 by cysLT1 receptor stimulation increases eosinophiles transendothelial migration and superoxide generation [42]. 

In paraquat poisoning, MK reduces the plasma concentration of TNF alpha and inhibits neutrophil infiltration [43]. In doxorubicin poisoning, MK also reduces TNF-α and IL-1β levels [44]. The level of caspase-3 and proinflammatory interleukins IL-1β and IL-17 is also reduced by MK [45,46]. In the case of experimental fibrosis induced with bleomycin, both zileuton (an inhibitor of 5-lipoxygenase) and MK-571, a peptide leukotrienes receptor antagonist, reduce long injury and collagen deposition but cause neutrophil infiltration of the lung. Our results agree with the experimental data that show an increase in the thickness of the interalveolar septum in diabetes [47]. In patients with sarcoidosis, MK reduces the proliferation of pulmonary myofibroblasts and decreases the expression of TGF beta. MK has a protective effect on the lungs by reducing the release of proinflammatory cytokines from macrophages [48].

The obtained data are in agreement with the results of some clinical studies that show that MK reduces pulmonary inflammation and neutrophil infiltration in children with cystic fibrosis [49] and with the results showing that MK reduces neutrophil infiltration in different organs in pathological conditions [50].

Our results are in agreement with the data showing that the W/D lung ratio is increased in diabetic animals compared to normal rats. DM causes an increase in vascular permeability associated with an increased release of proinflammatory cytokines [51,52]. This causes an increase in vascular extravasation and an increase in the W/D lung ratio in animals with experimental diabetes. Our data are in agreement with data showing that MK reduces lung vascular permeability [48,49,53,54]. In other diseases such as Raynaud’s syndrome, MK reduces vascular permeability, decreases fluid extravasation, and also reduces thermoalgesia [55].

The weight loss of diabetic animals was attenuated by the administration of MK.

MK has a protective effect not only in diabetic lung lesions but also a partial protective effect against testicular, liver, and kidney diabetic damages [56]. Experimental studies have shown that this drug has a protective effect in other pathological situations as well, such as pyelonephritis, gastric ulcer, and cisplatin-induced experimental acute renal failure [57,58,59,60].

Pulmonary fibrosis, regardless of its cause, is a difficult problem in clinical practice. In idiopathic pulmonary fibrosis, treatment with prednisone or a combination of prednisone and azathioprine is used. Sometimes, N-acetylcysteine is also administered in clinical practice [61]. Pirfenidone and nintedanib are two drugs recently introduced in the treatment of this type of pulmonary fibrosis [62]. The mechanisms of action of the two drugs are different from those of MK. Pirfenidone stimulates the activity of some collagenases and reduces the synthesis of TNF alpha and IL-6. Nintedanib is an intracellular tyrosine kinase inhibitor. This drug also reduces the activity of other factors involved in the development of fibrosis, such as vascular endothelial growth factor and platelet-derived growth factor [63]. These drugs have not been used in the treatment of diabetic pulmonary fibrosis. The existing data show that these drugs reduce (in different proportions) the development of pulmonary fibrosis, but they can only partially solve the problem. MK has a different mechanism of action from all the drugs mentioned previously and an effect of reducing pulmonary fibrosis that could be clinically useful as well.

## 5. Conclusions

The obtained results showed that MK significantly reduced pulmonary fibrosis and the pulmonary lesion score in experimental diabetes. Our data suggest only as a hypothesis the possibility of associating MK with antidiabetic therapy to reduce the development of pulmonary fibrosis. Blood sugar correction in these patients is always recommended, but it does not solve the problem of pulmonary fibrosis. Recent experimental studies have shown that the administration of vitamin D3 in rats with experimental diabetes reduces the development of pulmonary fibrosis [64]. The mechanism of action of vitamin D3 in this case is completely different from the mechanism of action of MK. Another direction of research related to the treatment of pulmonary fibrosis is the administration of anticorisin monoclonal antibodies [65]. Corisin is a proapoptotic peptide involved in the exacerbation of pulmonary fibrosis. Compared to these monoclonal antibodies, MK would have the advantage of a wide experience of use in humans. Of course, future studies are needed regarding the doses of MK needed to reduce the development of diabetic pulmonary fibrosis in humans. It is also necessary to determine if the use of this peptide leukotriene antagonist in diabetic children can prevent the development of pulmonary fibrosis.

### Limitations

A limitation of the study is the absence of plasma concentration determinations of leukotrienes.

## Figures and Tables

**Table 1:** TAS values in all animal groups included in study (results are in Tolox units).

Group	Initial	*p*	After 72 h	*p*	p*	After 8 Weeks	*p*	p*
Control	41.85 ± 2.71	NS	40.18 ± 1.46	<0.01	NS	40.08 ± 2.11	<0.01	NS
MK	39.55 ± 1.23	NS	37.65 ± 2.32	<0.01	NS	38.76 ± 2.41	<0.01	NS
STZ	39.89 ± 1.03		23.42 ± 2.03		<0.05	25.52 ± 2.09		<0.01
STZ+MK	40.44 ± 2.09	NS	27.45 ± 1.24	<0.05	<0.05	33.29 ± 1.64	<0.01	<0.05

*p* versus STZ group, p* versus initial.

**Table 2:** SOD values in all animal groups included in study (results are in U/mL).

Group	Initial	*p*	After 72 h	*p*	p*	After 8 Weeks	*p*	p*
Control	129.5 ±18.3	NS	133.2 ± 15.7	<0.01	NS	128.4 ± 13.8	<0.01	NS
MK	135.1 ± 16.8	NS	129.6 ± 16.5	<0.01	NS	132 ± 10.5	<0.01	NS
STZ	134.4 ± 19.5		76.1 ± 14.3		<0.01	77.2 ± 11.9		<0.01
STZ+MK	130.7 ± 17.7	NS	91.3 ± 12.9	<0.05	<0.05	95.7 ± 13.3	<0.05	<0.05

*p* versus STZ group, p* versus initial.

**Table 3:** MDA values in all animal groups included in study (results are in microM/L).

Group	Initial	*p*	After 72 h	*p*	p*	After 8 Weeks	*p*	p*
Control	1.37 ± 0.13	NS	1.39 ± 0.14	<0.01	NS	1.43 ± 0.10	<0.01	NS
MK	1.41 ± 0.16	NS	1.42 ± 0.09	<0.01	NS	1.44 ± 0.13	<0.01	NS
STZ	1.36 ± 0.12		2.67 ± 0.2		<0.01	2.72 ± 0.22		<0.01
STZ+MK	1.42 ±0.11	NS	2.11 ± 0.17	<0.05	<0.05	2.07 ± 0.15	<0.0.5	<0.05

*p* versus STZ group, p* versus initial.

## Data Availability

The original contributions presented in the study/Supplementary Material, further inquiries can be directed to the corresponding author.

## Supplementary Materials

The following supporting information can be downloaded at: https://www.mdpi.com/article/10.3390/medicina60050749/s1, Figure S1. Control group, normal lung tissue (×100). Figure S2. Control group, with thin alveolar walls, uniform in size and shape (×400). Figure S3. STZ group (after 8 weeks), perivascular inflammatory infiltration (×100). Figure S4. STZ group (after 8weeks), alveolar septum thickening, septal inflammatory cell infiltration (×400). Figure S5. MK+STZ group (after 8 weeks), the alveolar septum thickening was significantly reduced (×100). Figure S6. MK+STZ group (after 8 weeks), reduced scattered interstitial haemorrhage, reduced thickening of the alveolar septum.
