# Supplementary material for: Montelukast Influence on Lung in Experimental Diabetes
Source: Medicina (Kaunas). 2024 Apr 30;60(5):749. doi: 10.3390/medicina60050749 (PMC11123472; doi:10.3390/medicina60050749)
Supplement: Supplementary file 1 [file medicina-60-00749-s001.zip › medicina-2925628-supplementary.pdf]

Supplementary Materials

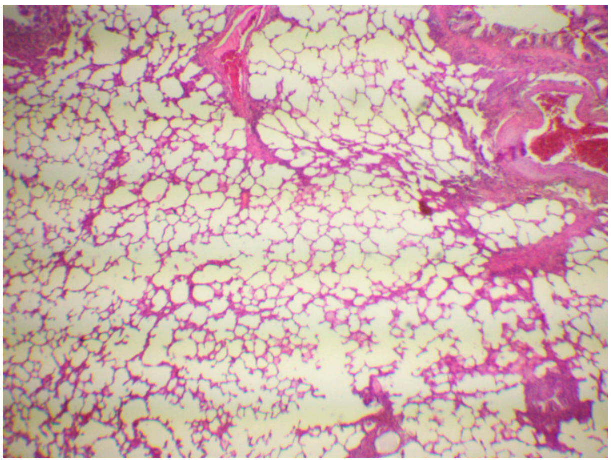

Figure S1. Control group, normal lung tissue (x100)

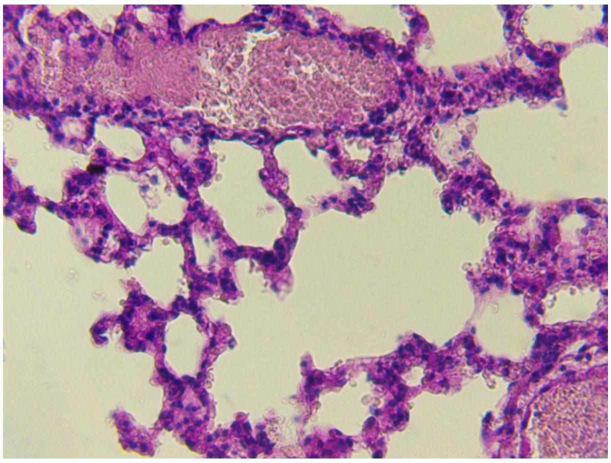

Figure S2. Control group, with thin alveolar walls, uniform in size and shape (x400)

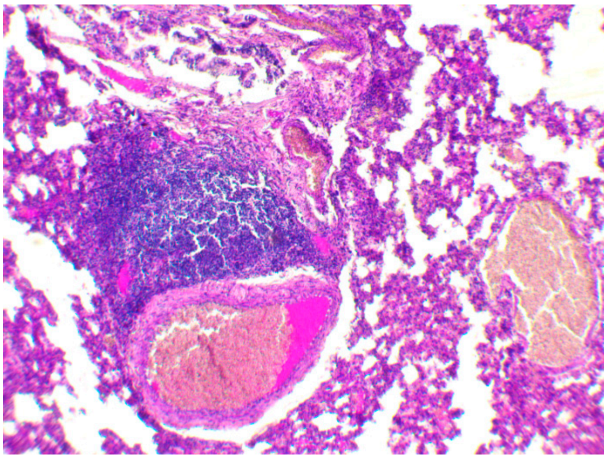

Figure S3. STZ group (after 8 weeks), perivascular inflammatory infiltration (x100).

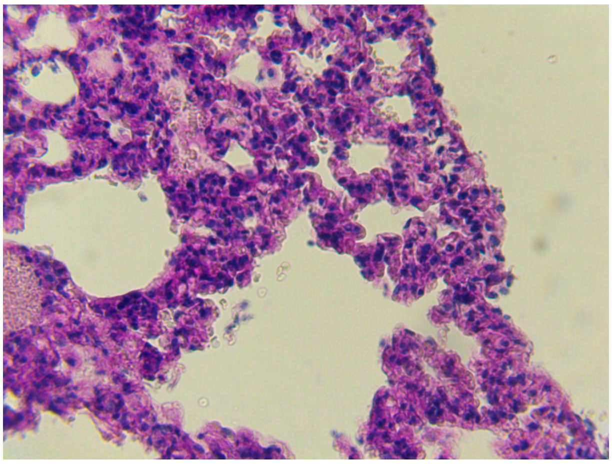

Figure S4.STZ group (after 8weeks), alveolar septum thickening, septal inflammatory cell infiltration (x400).

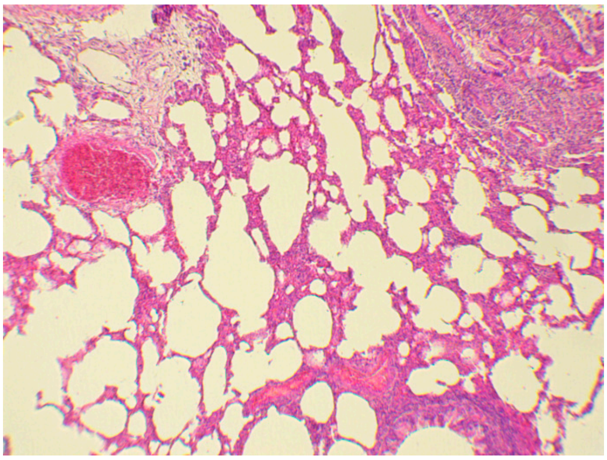

Figure S5.MK+STZ group(after 8 weeks), the alveolar septum thickening was significantly reduced (x100)

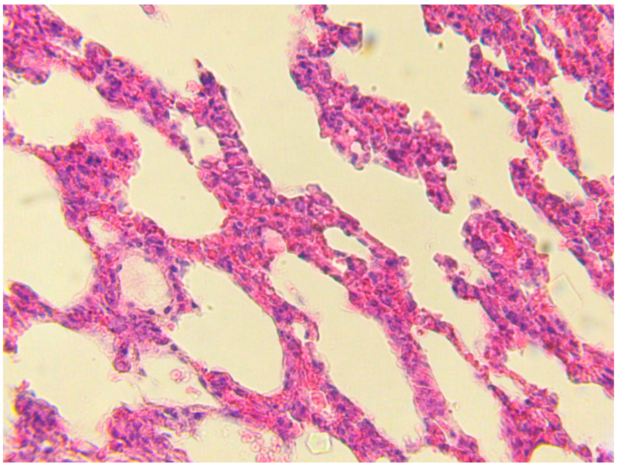

Figure S6. MK+STZ group(after 8 weeks), reduced scattered interstitial haemorrhage , reduced thickening of the alveolar septum
